# GPI-AP: Unraveling a New Class of Malignancy Mediators and Potential Immunotherapy Targets

**DOI:** 10.3389/fonc.2020.537311

**Published:** 2020-12-04

**Authors:** Nada H. Hussein, Nada S. Amin, Hend M. El Tayebi

**Affiliations:** Molecular Pharmacology Research Group, Department of Pharmacology and Toxicology, Faculty of Pharmacy and Biotechnology, German University in Cairo, Cairo, Egypt

**Keywords:** glycosylphosphatidylinositol, glycosylphosphatidylinositol-anchored protein, immunotherapy, cancer, immunology

## Abstract

With millions of cases diagnosed annually and high economic burden to cover expensive costs, cancer is one of the most difficult diseases to treat due to late diagnosis and severe adverse effects from conventional therapy. This creates an urgent need to find new targets for early diagnosis and therapy. Progress in research revealed the key steps of carcinogenesis. They are called cancer hallmarks. Zooming in, cancer hallmarks are characterized by ligands binding to their cognate receptor and so triggering signaling cascade within cell to make response for stimulus. Accordingly, understanding membrane topology is vital. In this review, we shall discuss one type of transmembrane proteins: Glycosylphosphatidylinositol-Anchored Proteins (GPI-APs), with specific emphasis on those involved in tumor cells by evading immune surveillance and future applications for diagnosis and immune targeted therapy.

## Introduction

Cancer is one of the most aggressive diseases responsible for thousands of deaths annually and millions newly diagnosed (1). The major problems are family history, unhealthy lifestyle, late diagnosis, and the detrimental side effects of chemotherapy and radiotherapy (2). Consequently, thanks to rigorous research, scientists were able to develop characteristic hallmarks for transformation of normal cell into cancerous cell, with classic hallmarks published in 2000 (3). Classical hallmarks include apoptosis resistance to sustained growth, metastasis and angiogenesis (3). Lastly, increased telomerase activity empowers the tumor cell against senescence (3). Decade later, research suggested the involvement of additional hallmarks in cancer pathogenesis (4). Next-Generation Cancer hallmarks include metabolic reprogramming, escaping immune surveillance, tumor promoting inflammation and genome instability (4).

**Figure 1:**
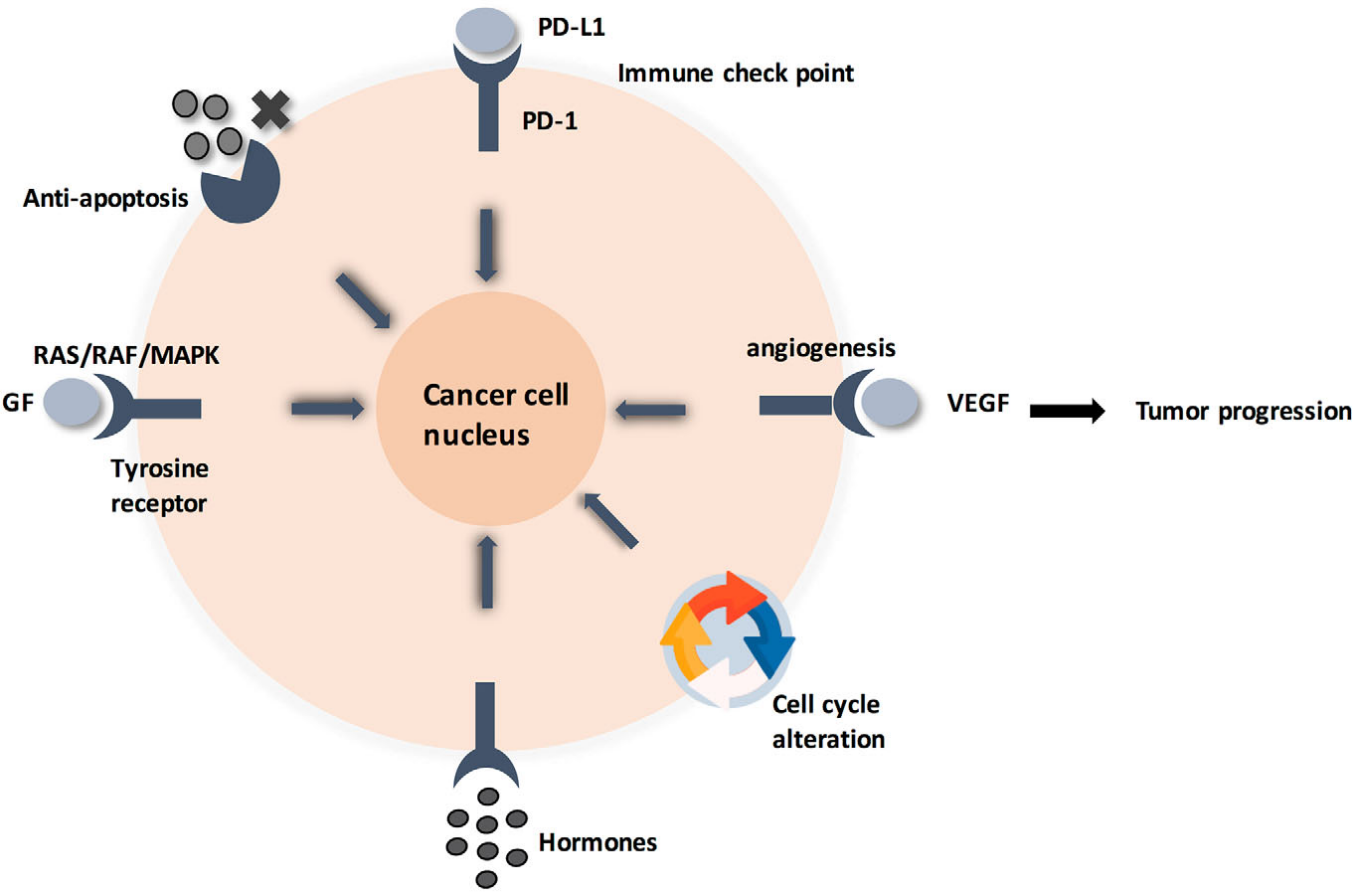
Cancer cell circuit. When treating cancerl as a minimalist/reductionalist view, tumor tissue consists of parenchyma and stroma that contain distinct cell types and subtypes that collectively enable tumor growth and progression. These cells communicate via signaling molecules, which are received by membrane anchored receptors, followed by pathway cascade to support tumor progression.

Zooming in, the detailed molecular pathway for each hallmark showed that all pathways are initiated by ligands binding to membrane-anchored proteins, thus activating a signaling pathway (4). This alters the expression of certain genes, leading to cellular response (**Figure 1**). Specifically, membrane-anchored proteins are either peripheral or integral. Peripheral proteins are superficially attached to the cell membrane. However, integral proteins have their heads exposed to extracellular matrix, and the tail is embedded in the phospholipid bilayer. Integral membrane proteins are further classified into Type-I transmembrane, Type-II transmembrane, Type-III transmembrane, Type-IV transmembrane, Type-V GPI-anchored proteins, and Type-VI transmembrane (**Supplementary Figure 1**) (5, 6). Consequently, predicting the type of a membrane-anchored protein is a challenging task due to the complexity of biosynthesis process. Specifically, biosynthesis process begins with joining early polypeptide chain and transmembrane area components in the ER, followed by recognizable proof and appropriate plan of TM areas, and lastly by reconciliation of TM spaces into the lipid bilayer (7, 8).

Based on the above information, studying membrane-anchored proteins is crucial in order to understand carcinogenesis and, therefore, deduce appropriate diagnosis and personalized therapy. In this review, we shall focus on Type-V GPI-anchored proteins (GPI-APs). We specifically chose this class of membrane-anchored proteins due to the expansion of investigations depicting their immune-modulatory role in cancer and current developments in immune targeted therapy. Briefly, GPI biochemical pathway consists of three phases: GPI anchor synthesis, joining protein moiety to GPI anchor, and final remodeling (9). Most of the literature emphasized on the role of GPI-APs in neurological and congenital disorders (10). However, only a few of the numerous examples of GPI-APs were studied for their role in cancer pathogenesis. For instance, CD-55 and CD-59 are crucial complement regulatory proteins elevated in various cancers, while Carcino-embryonic antigen (CEA) is a highly specific cancer biomarker, whose targeted immunotherapies are still in the early clinical trials (11). CEA mediates metastasis through binding to selectins in colon carcinomas (11). In addition, Mesothelin (MSLN) and Glypican-3 (GPC-3) are oncogenic GPI-APs over-expressed in multiple tumors, whose targeted immunotherapies are showing promising results in clinical trials (12). Specifically, MSLN promotes cancer proliferation and apoptosis resistance through NF-kB activation, while GPC-3 promotes cancer proliferation through sulfatase/Wnt- signaling pathway (12).

Therefore, this review highlights links between GPI-APs and GPI biochemical pathway. This represents a huge opportunity for molecular targeting whether by blocking GPI-AP as a whole or by blocking members of GPI pathway, unlike other oncogenic proteins that have only few molecular targets. This is because GPI pathway involves 26 genes and at least 150 proteins are confirmed as GPI-APs (9). Moreover, our review sheds light on the use of GPI-APs and GPI pathway members as cancer biomarkers. The outline of our review starts with GPI pathway definition, followed by an insightful discussion of examples of established GPI-APs; highlighting pros and cons of immune targeted therapies and possible future applications.

## GPI-Anchored Proteins

### Discovery

GPI proteins were first discovered in 1963 through studying selective cleavage of Alkaline Phosphatase by bacterial phospholipase-C (PL-C). Two decades later, hypothetical GPI-AP structure was confirmed by isolating actual GPI-APs, such as acetylcholinesterases, from different organisms (13, 14). Finally, variant surface glycoprotein (VSG) ectopic expression in African trypanosome system was used as a model to dissect the GPI biochemical pathway and GPI-linked proteins/glycoproteins characteristics (15).

### Structure

Based on the substance to be anchored, GPIs are currently classified into protein-linked GPIs and non-protein-linked GPIs. Protein-linked GPIs consist of protein or glycoprotein attached to GPI anchor, while non-protein-linked GPIs consist of GPI anchor and non-protein extracellular glycol conjugates (13–15). All GPIs share a common structure, starting with a lipid tail attached to cell membrane, followed by glycan core, and finally anchored substance. Zooming in, the lipid tail consists of a lipid moiety attached to the inositol ring by a phosphodiester bridge. Depending on the organism, there are various types of lipid moieties such as ceramide (slime mold proteins), diacylglycerol (protozoa), and 1-alkyl-2-acylglycerol (most mammalian proteins) (11–13). The conserved glycan core consists of Manα(1→2)Manα(1→6)Manα(1→4)GlcNH2α(1→6)-myo-Inositol-1-PO4-lipid. Finally, the C-terminus of polypeptide chain is conjugated to the 6-O-position of the non-reducing-end mannose of GPIs through a phosphoethanol amine group (P-OEtNH2) (14, 15) (**Supplementary Figure 2**).

### Characteristics/Identification

GPI-APs feature a NH2-terminal signal peptide and a COOH-terminal GPI signal peptide, where these peptides can be utilized for computational examination to forecast whether a certain gene produces a GPI-anchored protein or not, such as the Web-based “enormous P indicator” calculation (11, 12). Another key component is affectability of GPI-APs to phospholipases, which can be applied in different tests for recognizing GPI-APs, like the migration of proteins from the pellet to the supernatant after treating cells with PI-PLC. Moreover, PI-PLC cleavage produces cross-responding determinant inositol-1,2-cyclic monophosphate; consequently, applying immune-based assays to detect the cross-reacting determinant serves as a test to identify GPI-AP. It should be noted, however, that some GPI-APs are impervious to PI-PLC cleavage due to C-2 inositol acylation. On the other hand, all GPI-anchored proteins are labile to serum GPI-phospholipase-D (GPI-PLD). Cleavage by GPI-PLD, however, does not produce “cross-responding determinant.” Instead, it produces the inositol acyl gathering (one unsaturated fat connected to the protein) which may avoid total Triton X-114 Phase separation after GPI-PLD digestion, therefore yielding false-negative results for GPI proteins identification by Triton X Phase separation test (13–16).

GPI-APs can additionally be traced by radioactively labeled constituents, such as [3H]myo-inositol, [3H]mannose-, [3H]glucosamine-, and [3H]inositol. Lastly, a unique chemical test for GPI-APs is deamination of glucosamine moiety by nitrous acid, which results in a very specific cleavage at the glucosamine-inositol glycosidic bond. The products of this reaction are a phosphatidyl inositol part, and a free reducing end on the GPI glycan (2,5-anhydromannose), where the former can be identified by solvent partition coupled to mass spectrometry. Meanwhile, the sugar end is additionally reduced to [1-3H]2,5-anhydromannitol (AHM) by sodium borotritide. AHM is then attached to a radiolabel or fluorophore to be assayed by different identification techniques, such as sequencing, after treatment with exoglycosidases and tandem mass spectrometry (13).

The last characteristic is endocytic targeting, by which, like all extracellular proteins, they are downregulated and degraded by endocytosis. In fact, the proof demonstrates that, in spite of lacking a cytoplasmic tail, GPI-APs can be degraded by clathrin-dependent endocytosis. This paradox has been unraveled by studying clathrin-dependent endocytosis of PrP and the GPI-anchored urokinase plasminogen activator receptor (uPAR), where internalization was achieved by association with the transmembrane LDL receptor-related protein. Other mechanisms of endocytosis for GPI-anchored proteins exist, where GPI-APs undergo endocytosis through caveolae, a gathering of lipid pontoons coated with caveolin. Yet, this claim has been nullified for different reasons. Firstly, GPI-APs are poor in caveolae. Additionally, regular endocytosis requires cutting of the endocytic vesicle from the cell membrane by a GTPase, dynamin which is repudiated by different investigations. Likewise, it is proposed that GPI-anchored proteins are broken down by a pathway called GPI-anchored protein-advanced early endosomal compartments (GEEC) pathway. Finally, a novel pathway involving the raft protein—Flotillin-1—is suggested for GPI-APs endocytosis (13–15).

### GPI Anchoring Pathway

**Figure 2:**
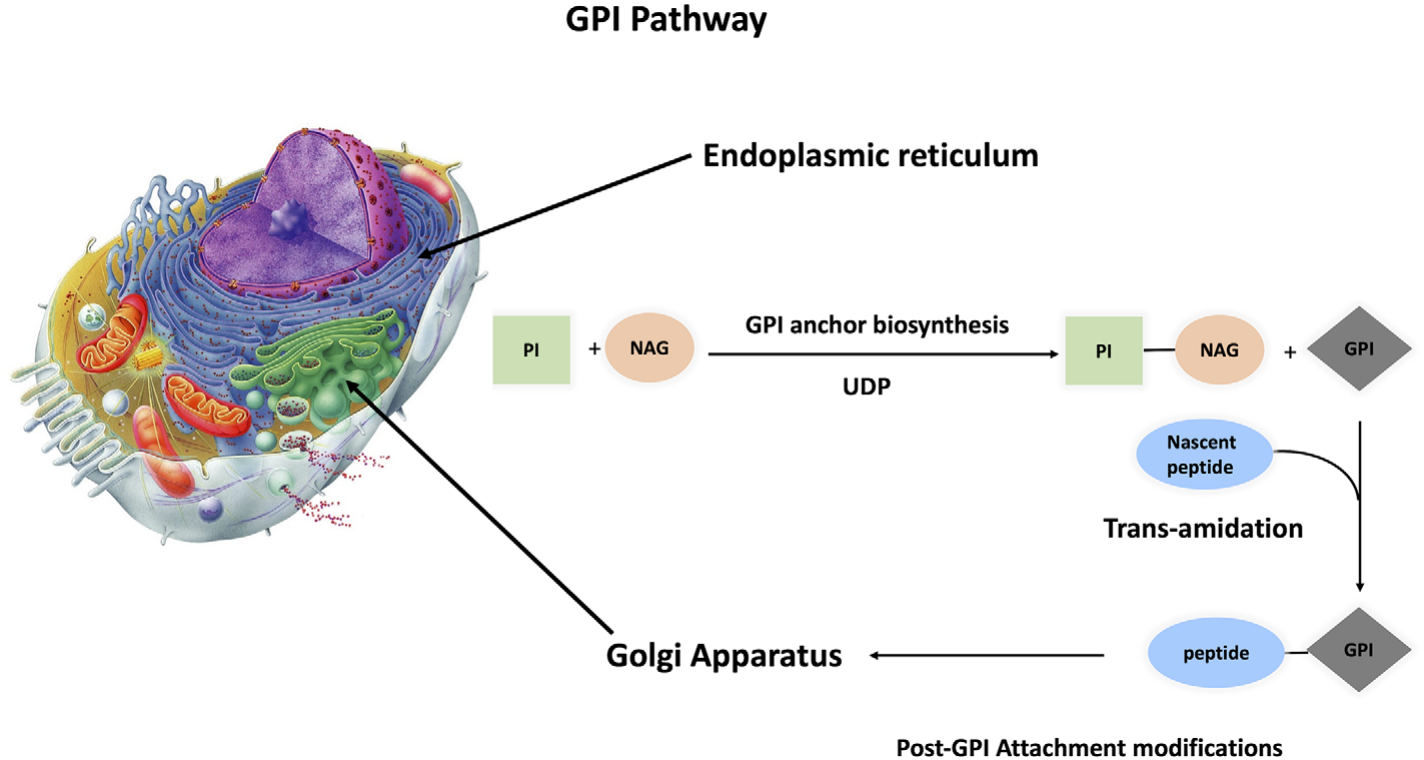
Synthesis of GPI anchored protein GPI anchoring pathway is divided into three steps, GPI anchor biosynthesis followed by transamination (protein attachment to GPI anchor). These two steps take place inside the ER, and with the aid of p24 protein family, the primary GPI-AP is transferred to the Golgi apparatus for post attachment modifications. Finally, mature GPI-AP is exported to cell membrane.

The conserved glycan core of GPI anchors suggests a generally conserved biosynthetic pathway among different species (13). This is a crucial pathway because a minimum of 150 different human proteins are anchored to the extracellular layer of cell membrane via glycosylphosphatidylinositol (GPI). GPI-AP synthesis is divided into three parts: biosynthesis, protein attachment to GPI, and GPI-AP remodeling. This complex process is performed in 15 steps, involving 26 genes that code for 15 enzymes, with twenty-two phosphatidyl inositol glycan (PIG) genes responsible for biosynthesis and polypeptide chain attachment to GPI, while four post-GPI attachment to proteins (PGAP) genes are responsible for GPI modifications (16–20) (**Supplementary Table 1** and **Figure 2**). The roles of these genes were tested by gain-and-loss-of-function experiments in different organisms, impacting the capability of protein attachment to plasma membrane regardless of expression level inside the cell.

### Signaling Functions

GPI-anchored proteins are involved in multiple cellular functions as shown in **Supplementary Table 2**. First of all, analogous to bacterial phospholipase-C, phospholipases exist endogenously in order to release a protein from the membrane in response to a stimulus. The detached protein may have the same or slightly different functions compared to the membrane-anchored precursor. Interestingly, several GPI-anchored proteins are found to be secreted as well as having cross-reacting determinant, which further supports PI-PLC cleavage. Other enzymes are suggested, such as GPI-specific phospholipase-D, which is present in the blood and has proved promising activity *in vitro*. Notch is a Wnt signaling inhibitor, has also been proven to cleave the GPI anchor of many proteins such as glypican family, which regulate Wnt signaling. Finally, angiotensin-converting enzyme (ACE) is a promising option that is still under study. In conclusion, these enzymatic reactions result in controlled release of GPI-AP (13–15, 17).

GPI pathway is additionally responsible for anchoring the molecule of interest to membrane microdomains called lipid rafts, which are rich in sphingomyelin, glycosphingolipids, and cholesterol. Lipid rafts serve as compartments for myriads of functions such as endocytosis and cell signaling. GPI proteins are enriched in lipid rafts due to favorable hydrophobic interactions between saturated GPI anchor fatty acid chains and lipid raft-resident membrane lipids forming a more tightly packed gel-like phase than the surrounding semifluid phospholipid bilayer. As a result, lipid rafts are partially resistant to nonionic detergents, which serve as a base for Triton X Phase separation test described in the Identification section (13–15).

Furthermore, GPI signal sequence is used to determine whether the protein is placed in the basolateral or apical side of the membrane, as demonstrated by multiple studies. A signal peptide for either the folate receptor or PrP was conjugated to the C-terminus of green fluorescent protein (GFP), and then the cells were imaged. Interestingly, the GFP-folate receptor fusion was localized in the apical surface, while GFP-PrP fusion was localized in the basolateral surface. Of note, it is speculated that the surrounding lipid environment plays a role in this sorting (14, 15, 18).

Finally, binding of GPI-AP to its antibody causes a rise in intracellular Ca^2+^, tyrosine phosphorylation, proliferation, cytokine induction and oxidative burst, triggering signal transduction pathways. This was illustrated by replacing GPI anchor with a transmembrane domain, which abolished the signaling cascade. This is supported by further evidence, where enrichment of signaling molecules coupled to GPI-anchored proteins is critical for lipid rafts signaling function. However, the mechanism of how GPI anchor transduces signals is not fully understood yet. One possible explanation is direct attachment of GPI-AP to signaling transmembrane proteins within lipid rafts. This was proved by replacing the GPI anchor motif of differentiation-promoting neural cell adhesion molecule (NCAM) with the signal sequence from carcinoembryonic antigen (CEA), producing a hybrid protein with NCAM ectodomain, but CEA-like antidifferentiation activity (10). Another explanation is that these antibody-induced signaling events depend on the induction and coalescence of lipid raft nanoclusters. Accordingly, GPI-APs function as hydrolytic enzymes, receptors, adhesion molecules, complement regulatory proteins, and other immunologically important proteins, many of which are implicated in many diseases (14, 15, 17).

### GPI Mutations in Diseases

Mutations of GPI pathway genes are well documented in neurological and congenital disorders (9, 10). To start with, clinical data of 152 individual patients was reviewed and compared against the phenotypic information obtained from Human Phenotype Ontology (HPO). Statistically significant difference was observed between the GPI pathway genes and frequencies of phenotypes in the musculoskeletal system, cleft palate, nose phenotypes, and cognitive disability (21). These phenotypes are congruent with inherited GPI deficiencies (IGDs) (21). Getting deeper, non-synonymous variant (c.968A > G) in the seventh exon of GPAA1 causes inherited vascular anomalies (VAs). VAs comprise a wide spectrum of abnormalities, from blood vessels, to angiogenesis in different tumors subtypes due to variation in multiple tyrosine kinase (TK) receptor signaling pathways, such as TIE2, PIK3CA and GNAQ/11 (22). Another example, PIG-O biallelic variation, was observed in Mabry disease (23). Mabry disease symptoms include intellectual disability, distinctive facial features, intractable seizures, and hyperphosphatasia (23). In addition, compound heterozygous variants in PIG-S:c.148C > T and c.1141_1164 resulted in infantile spasms (ISs), severe global developmental delay, hearing loss, visual impairment (cortical blindness), hypotonia, and intellectual disability (24). Furthermore, two subsets of mutations were associated with early infantile epileptic-dyskinetic encephalopathy; first is the homozygous c.384del variant of PIG-P gene (25), which led to the frame shift of 6 codons before the expected stop signal (25), and second are PIG-Q mutations, particularly PIG-Q novel variants, which included two missense mutations (p.G17R; p.G449R), a canonical splice site substitution (c.942+1G>A), an in‐frame deletion (p.A377_S389del) and three frameshifts (p.Q527Afs*75, p.R538Afs*24 and p.G557Dfs*) (26). Finally, mutational analysis of PIGA identified 124 PIG-A mutations in 92% of paroxymal nocturnal hemoglobinuria (PNH) patients, of which 101 were distinct mutations and 23 were recurrent (27).

Some studies tested the impact of GPI pathway manipulation on dependent GPI-anchored complement regulatory protein, CD-59, which was found to be under expressed in congenital and neurological disorders, as well as PNH (21–27). CD-59 was, however, overexpressed in most solid tumors (28). Weighted gene co-expression network analysis (WGCNA) was used to identify the hub gene in Kirsten-rat sarcoma viral oncogene homolog (KRAS) mutant colorectal cancer (CRC) patients (29). Surprisingly, PIG-U was under expressed in KRAS mutant CRC compared to normal controls (29). Specifically, KRAS mutant patients had a poor prognosis and PIG-U low expressing samples show elevated MAPK signaling activity (29). Contrastingly, our recent work showed that PIG-C is overexpressed in breast cancer (30). Also, PIG-C SNP was observed in HCC (31). Moreover, gain of chromosome copy number in breast cancer results in elevated expression of transamidase subunits such as GPI anchor attachment protein 1 (GPAA1) and GPI class T (PIG-T) (32). Therefore, more studies are required to depict the role of GPI pathway in cancer. In the next section, functions of GPI anchored proteins and immune therapies will be discussed in detail.

## GPI-Anchored Proteins in Cancer Immunomodulation And Targeted Therapy

### Complement Regulatory Proteins: CD55 and CD59

#### Structure and History

Complement regulatory proteins are CD-46, CD-55, and CD-59. Our scope includes the GPI-APs: CD-55 and CD-59. CD-55 is called Decay Accelerating Factor (DAF), while CD-59 is called Membrane Attack Complex Inhibitor (MAC-i). CD-55 has an extracellular domain that is composed of 4 short consensus repeat (SCR) domains (33). CD-59 is a LY-6 like protein, with molecular weight 18-20 KDa (34).

#### Pathway and Function 

Complement system is a powerful soldier in innate immune system attack, which serves as first defense line against infections. DAF is a complement regulatory protein (CRP). CRPs are crucial in order to keep nearby normal cells safe from bystander killing and complement-mediated damage. CD55 accelerates the dissociation and decay of C3 convertases (C4bC2a and C3bBb) and in turn the C5 convertases into their building units, after which they are no longer able to rejoin. Of note, DAF does not work in a proteolytic manner (35–37). Specifically, CD55 is a ligand for CD97, where CD55 binds to CD97 at EGF domain region (37–39). The EGF domain requires calcium to maintain conformational stability, making cells highly resistant to complement activation pathway (39). Consequently, this enables cancer cells to escape immune system attack.

Similar to CD55, MAC-i is a complement regulatory protein. After CD46 and CD55, CD59 acts as last line of defense against complement activation, where it sequesters C8 and C9 components, therefore preventing C9 polymerization into the pore-forming membrane attack complex (40). Specifically, CD59 binds to the α-chain of C8 and to the β domain of C9. In an attempt to decipher CD-59 molecular pathway, Murray and Robins tested the proteins that underwent phosphorylation upon stimulation of CD59 in THP-1 and U937 hematopoietic cell lines (41). Indeed, protein tyrosine kinase family (Src) phosphorylation was observed upon CD59 activation (41). Sequentially, Src resulted in tyrosine phosphorylation of the adaptor proteins p120cbl and Shc, the cytoplasmic non receptor tyrosine kinase Syk, and its close relative Zap-70 (41). Interestingly, interaction of Phospho-Syk with Grb2 induced the MAP kinase (ERK1/ERK2) pathway (42). Moreover, the study observed inositol-5- phosphatase SHIP (43), in immunoprecipitations of Shc upon CD59 activation. SHIP phosphorylation appears to be triggered by various growth factors and cytokines (43) and may be involved in apoptosis and growth regulation (44).

#### Expression Level 

Elevated CD55 is correlated with poor prognosis of colorectal, breast, pancreatic, gallbladder (IHCC), and gastric cancers. Paradoxically, CD55 expression is decreased in ovarian cancer and lung cancer, with certain SNPs associated with higher risk such as rs2564978 variant and rs2564978 (36, 37, 39, 45). As for CD59, it was found to be overexpressed in head and neck, colorectal, ovarian, (28, 46, 47), and in cervical cancer as well (48). In these cases, overexpression appears to be correlated to poorer prognosis (28, 46, 47).

#### *In Vivo* Model 

In terms of immune-based therapy, antibodies raised against CD55 did not show overall consistent results; while 791T showed marvelous results in preclinical trials when conjugated to ricin-A chain, it failed phase I clinical trials. It was believed that therapeutic antibodies against CD55 should target SCR-3, while the use antibodies targeting other SCRs should only be restricted to immune-based assays. Until 2017, a patent illustrated that 791T antibody binds to epitopes located in SCR1 and SCR2, therefore opening the door for using more epitopes to raise antibodies against DAF. Interestingly, 791T was used to raise 105 AD7, a human anti-idiotype monoclonal antibody that mimics CD55. In 2000, the use of 105 AD7 as a peptide vaccine was first reported. Indeed, it stimulated anti-tumor immune reactions in patients with HLA/ A1,3,24 and HLA/DR1,3,7 haplotypes. In 2005, Ziller et al. reported mini antibodies MB-55 (against CD55) and MB-59 (against CD59) that increased lysis of Karpas 422 and Hu-SCID1 lymphoma cell lines when combined with Rituximab. In 2007, Macor et al. reported the same findings in vivo using LCL2 lymphoma cells in female SCID mice (49–52).

Strategy of CD59 blockade is showing promising results, where ex vivo treatment of colorectal cancer patients’ T-cells with CD59-specific antibodies, MEM-43, and HC-1 has shown significantly enhanced antitumor immune response (53). Another study compared the effect of CD59 silencing on HT-29 cells viability when treated with 5-flurouracil or oxaliplatin. Indeed, silencing of CD59 enhanced the sensitivity of HT-29 cells to 5-fluorouracil and oxaliplatin (54). In addition, CD-20+-Lymphoma mice treated with MB-55 and MB-59 had improved response for rituximab immunotherapy, where 70% of mice survived when treated with rituximab and MB-59 or MB-55 combination, while 30% of mice survived when treated with rituximab alone (55). One possible clue is a study showing that CD55 and CD59 expression guards HER2‐breast cancer cells from trastuzumab-induced complement-dependent cytotoxicity (56). However, account should be taken as targeted CD-59 depletion in mouse embryonic cells resulted in mice with increased RBCs count and higher susceptibility to complement lysis (57).

#### Insights

CD-59 is a promising marker as demonstrated by non-invasive graphene oxide based immune sensor with detection range 1fg/ml to 10 ng/ml (58). However, therapeutic potential for CD-59 blockade in tumors still requires more testing *in vivo*. Current evidence supports adjuvant use of CD-59 blockade in order to enhance efficacy of anti-cancer immune therapies (54, 55). However, caution should be taken, as adverse CD-59 blockade may cause paroxysmal nocturnal hemoglobinuria (PNH), which is characterized by elevated complement activity, RBCs lysis and CD-59 decreased expression (59). One possible refutation is the study using CD59 knockout and CD-59/CD-55 double-knockout mouse models (60). Surprisingly, Crry (complement receptor 1 (CR1)–related gene), neither CD59 nor DAF, was indispensable for murine erythrocyte protection *in vivo* from spontaneous complement attack, despite murine RBCs sensitivity to antibody-induced complement lysis *in vitro*. This proves that C3 inhibition is more critical *in vivo* rather than C8 and C9.

On the brighter side, C5 inhibitor eculizumab showed success and improvements in some PNH case reports, owing to the restoration of CD-59 activity (61). Nevertheless, eculizumab still faces challenges, including persistent anemia with some patients requiring transfusions, and incomplete C5 inhibition with breakthrough hemolysis (61). This prompted the investigation of several second-generation C5 inhibitors (new mAb, siRNAs and small molecules) (62). Altogether, this ultimately justifies the rationale behind the use of CD-55 and CD-59 silencing as anti-cancer therapy, especially with establishment of CD-59 knocked out cancer cell lines which can be used for further in vivo investigations (63)

### (CD66) Carcinoembryonic Antigen (CEA)

#### Structure and History 

CEA was discovered in 1965 by Gold and Freedman as a surface protein found in gastrointestinal cancer cells. The human CEA family consists of 29 genes, out of which 18 are expressed. The expressed proteins are further classified into CEA subgroup (7 proteins) and pregnancy-specific glycoprotein subgroup (11 proteins) (11, 64). Our scope, once again, is GPI anchored CEAs: CEA-CAM 6 and CEA-CAM 5, which is also known as CD66e (11).

#### Pathway and Function

Due to unique sialofucosylated glycoforms within its GPI anchor, CEA was found to bind to L-selectin and E-selectin, therefore mediating cell adhesion. CEA-CAM 1 is tumor suppressing through decreasing cell proliferation and metastasis, while CEA-CAM-5 is tumor promoting through inhibiting colon cell differentiation, anoikis and apoptosis (65, 66). This is attributed to CEA-CAM-5 co-localization and subsequent activation of a5ß1 integrin signal transduction, triggering PI3K/AKT activity (67). CEA-CAM 6 oncogenic activity is due to mediating metastasis and resisting apoptosis. Increasing metastasis is through CEA-CAM 6 signaling induced SRC activity. SRC is a non-receptor Tyrosine kinase, which in turn phosphorylates focal adhesion kinase (FAK) and stimulates IGF-1 secretion. As a result, PI3K/AKT pathway is activated, consequently inducing epithelial mesenchymal transition (11, 68). Surprisingly, TGF-B/type II receptor (TBRII) interaction functions as a positive feedback, where TBRII forms a heterodimer with TBRI, which increases PI3K/AKT pathway activity and SMAD-3 phosphorylation. Phospho-SMAD-3 then forms a complex with Co-SMAD-4. This complex translocates to the nucleus and binds to CEA-CAM 6 gene promoter, elevating its expression (11).

#### Expression Level 

Stochiometric expression of CEA family members (CEA-CAMS 1,5,6) on epithelial cell guards normal tissue architecture through micro-environment interactions described above. Interestingly, CEA-CAM 1 is under expressed in CRC, breast cancer, prostate cancer and hepatoma (69). On the other hand, 70% of epithelial tumors over express CEA-CAM 6 rather than CEA-CAM 5 (11, 70, 71). Specifically, CD66 is elevated in colorectal, pancreas, liver, breast, ovary, and lung cancer (11, 70, 71). Interestingly, CEA-CAM 5 is temporarily produced during fetal development until birth. This encourages the use of CEA in diagnosis and targeted therapy.

#### In Vivo Models 

In 2015, Li et al. reported a bispecific antibody that targets CD3 and CEA. It was produced by genetically linking an anti-carcino embryonic domain (variable heavy H) to the C-terminal end of an anti-CD3 variable heavy, constant heavy 1, followed by co-transduction with anti-CD3 variable light-constant light in bacteria. The produced protein (antibody) is called S-FAB. To demonstrate *in vitro* efficacy of S-FAB, CEA-expressing human colorectal cancer cell lines HT29 and LS174T were exposed to human peripheral blood mononuclear cells (PBMCs) or isolated T-cells in the presence and absence of S-FAB. In absence of S-FAB, no cytotoxicity occurred, while treatment with S-FAB resulted in cytotoxicity, where cell viability was assayed using Cell Counting Kit (CCK) reagent. Accordingly, this study advanced to *in vivo* level, where NOD/SCID mice were subcutaneously injected with a mixture consisting of 1x10^6^ LS174T cells and 5 x106 fresh human PBMCs. An hour later, mice were IP injected with 20 μg S-FAB. Indeed, S-FAB successfully inhibited tumor growth, even better than anti-CD3 FAB (72). A similar bispecific antibody called CEA TCB has shown promising results *in vitro* and *in vivo* and is now tested in phase-I clinical trials (73). Other CEA-CAM targeting antibodies are MN-3, MN-14 and MN-15. Xenograft colorectal cancer mice showed higher survival, decreased adhesion to the extracellular matrix (ECM), and lower metastatic activity when treated with MN-3 and MN-15 (11, 71) However, these mAbs did not have a significant impact on tumor growth (11, 71). This called for testing other immune therapeutic strategies. For instance, By114-saporin is an immunotoxin tested on pancreatic xenograft model, where antibody mediated crosslinking increased apoptosis, enhancing the efficacy of saporin immunotherapy (74). Another study involved the use of Ab-IRDye where CEA antibody targets photosensitizer to cancer cells, thus, when cells are exposed to near-IR rays, oxidative burst occurred selectively in cancer cells causing their death, leaving normal cells intact. The efficacy of Ab-IRDye was tested *in vivo* using nude mice carrying gastric carcinoma xenograft (75). In addition, Silencing CEA-CAM 6 by RNAs decreased tumor proliferation by 68% in pancreatic cancer xenograft mice compared to control siRNAs (f. 88-90). Other observations were impaired angiogenesis, increased apoptosis, suppressed metastasis (0% treated versus 60% untreated, p=<0.05), and improved survival without toxicity.

#### Clinical Trials 

For details on clinical trials targeting CEA, **clinicaltrials.gov** was searched, and data were collected in **Table 1**. PANVAC-F (falimarev) vaccine and PANVAC-V (inalimarev) are therapeutic vaccines expressing CEA, where PANVAC-V is derived from vaccinia virus, while PANVAC-F is derived from fowlpox virus. In addition, a recent study reported first-generation CAR-T therapy against CEA. It was evaluated in phase-I clinical trials for CEACAM5+ lung cancer patients. Unfortunately, no significant clinical improvement was observed and the CAR-T cells lived for 14 days only. However, rising levels of systemic IFNγ and IL-6 indicate the presence of immune response when patients were pretreated with cyclophosphamide and fludarabine, as well as systemic IL-2 during CAR-T therapy. This means that CAR-T therapy targeting CEA is promising, but future studies should try to develop other CAR designs and T-cell production methods (76). A phase-I clinical trial was done to study the safety of anti-CEA second-generation CAR-T scfv-CD28/CD3z (Tandem), in which CAR-T therapy was delivered by infusion into hepatic artery. The results were promising; coadministration of IL-2 with CAR-T therapy has decreased CEA levels and increased serum IFNγ levels. Also, no patient suffered from severe adverse effects (77).

**Table 1:** CEA clinical trials.

NCT Number	Conditions	Interventions	Results
NCT00088413	Adenocarcinoma| Colorectal Cancer| Ovarian Cancer| Breast Cancer	PANVAC-V and PANVAC-F Plus Sargramostim	**For breast cancer** *Median OS=13.7 m. *Median PFS=2.5 m. **For ovarian cancer** *Median OS=15 m. *Median PFS=2 m. *1patient showed complete response *1patient showed 17% reduction in mediastinal mass *SE: mild injection-site reactions
NCT00408590	Ovarian Cancer| Primary Peritoneal Cavity Cancer	CEA-expressing measles virus|	*No dose limiting toxicities *Partial Disease :7/21 *stable disease:14/21
NCT00179309	Breast Cancer	PANVAC-V and PANVAC-F Docetaxel| Sargramostim	**For PANVAC +DOC.** *Median PFS= 6.6 m. *SE=23/25 **For DOC.** *Median PFS= 3.8 m. *SE=25/25 *SEs observed were anemia, tachyardia, and vomiting for DOX treated group.
NCT00103142	Colorectal Cancer| Metastatic Cancer	Falimarev| Inalimarev| Sargramostim| Autologous dendritic cells	For PANVAC +SARG. *recurrence free surv.= 55% For DC +SARG. *recurrence free survival= 47% *Immune response by T-cell against CEA was statistically similar between study arms.
NCT00645710	Liver Metastases| Recurrent Colon Cancer| Recurrent Rectal Cancer| Stage IV Colon Cancer| Stage IV Rectal Cancer	Anti-CEA mAb cT84.66| Gemcitabine hydrochloride| Floxuridine| Radiation: yttrium Y 90	**For cT84.66+Flox 0.10 mg/kg/Day:** *Median OS.= 23.2 m. **For cT84.66+Flox 0.15 mg/kg/Day:** *Median OS.= 73.2 m. **For cT84.66+Flox 0.20 mg/kg/Day:** *Median OS.= 41.2 m. (MTD) of HAI FudR=0.20 mg/kg/Day

OS, overall survival; PFS, progression-free survival; SE, side effects; MTD, maximum tolerated dose.

#### Insights

Finally, CEA has a unique expression pattern, making it a treasure for diagnosis using *in vivo* fluorescent imaging and immunosensing using nanomaterial-based electrochemical immunoassay, photoelectrochemical immunoassay, and optical immunoassay (78, 79). In addition, numerous studies combine CEA with other biomarkers for better diagnosis (80–83). As for prognosis, drop in CEA level can be used as a posttreatment watch (81, 82, 84). Clinical trials data prove the efficacy and safety of CEA-targeted immune therapy. However, they cannot be used as a single agent for treatment of different tumor types, where conventional chemotherapy is still needed to relieve tumor compact structure and add a synergistic tumor-killing effect. Finally, the use of anti-CEA as an adjuvant therapy decreased adverse effects compared to the use of standard therapies alone. More importantly, no serious adverse effects were observed in CEA-immune-based therapy.

### Glypican-3

#### Structure and History

Glypican-3 (GPC3) is a member of the glypican family, which are a group of GPI-APs. So far, 6 types (GPC1–GPC6) have been identified in mammals. Glypicans were shed in serum upon cleavage by Notum (a lipase for GPI anchors). Moreover, GPC3 has two heparan sulphate (HS) glycan chains attached to the C-terminus. GPC3 gene codes for a 70 kDa precursor core protein with 580 amino acids, which can be cleaved by furin producing a 40 kDa amino (N)-terminal protein and a 30 kDa membrane-bound carboxyl- (C-) terminal protein. Furin cleavage between Arg358 and Ser359 was found to be crucial for GPC3 activity in zebrafish, but not in HCC. It was also predicted that cleavage for GPI anchor occurs at serine 560 (85, 86).

#### Pathway and Function

**Figure 3:**
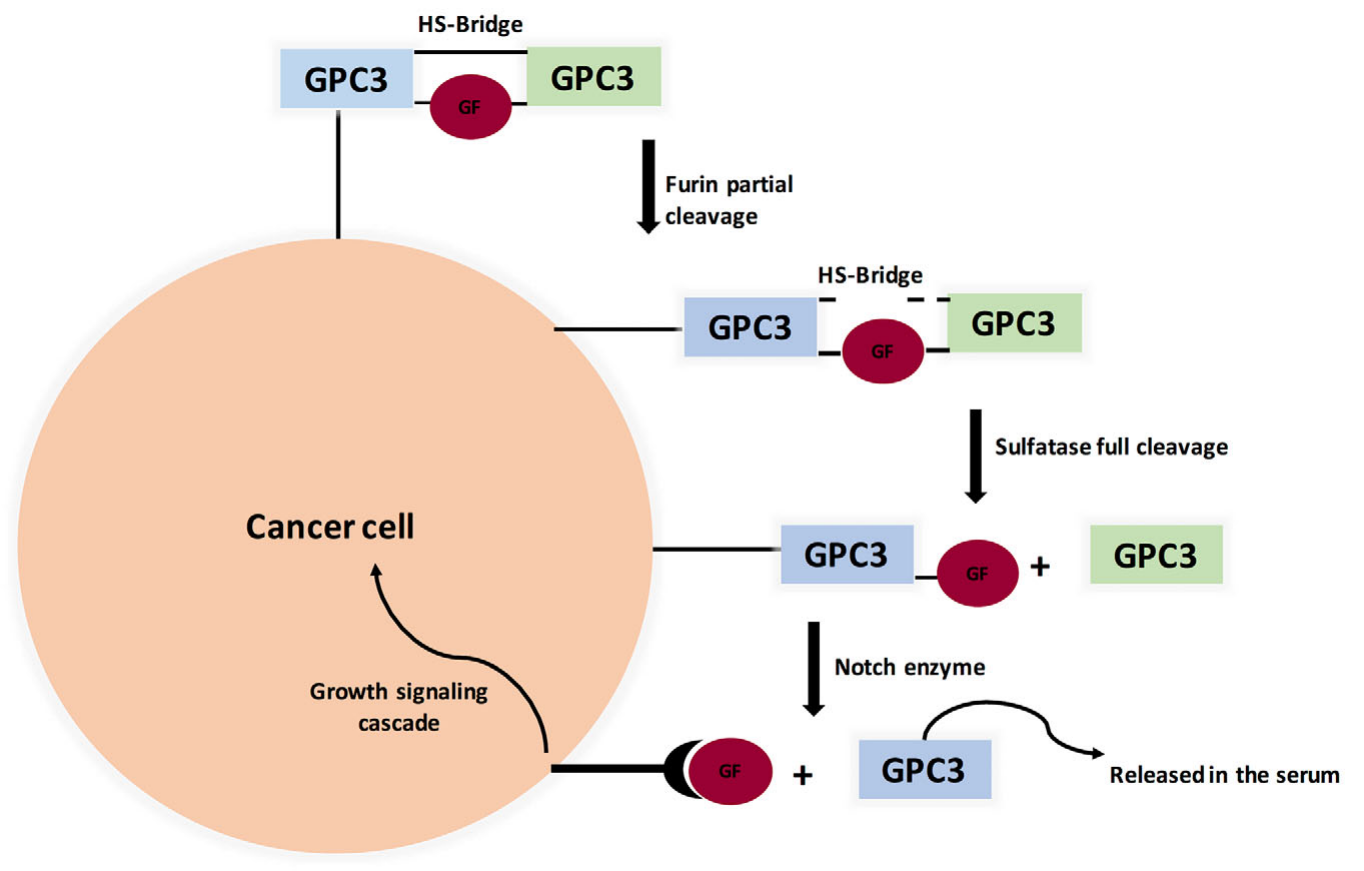
Proposed Model for Glypicans Due to Heparan Sulfate bridge (HS), GPC3 is not fully cleaved by Furin (unlike MSLN). Full cleavage is achieved when HS bonds are broken by Sulfatase 1 or Sulfatase 2. Interestingly, Sulfatase 1 has anti-oncogenic properties while Sulfatse 2 favors tumor progression by releasing growth factors, therefore binding to its cognate receptor and initiating signaling cascade. (GF=Growth Factor).

GPC3 knockout mice showed a distinctive phenotype: Simpson-Golabi-Behmel Syndrome (SGBS), an X-linked disorder characterized by pre- and postnatal overgrowth. This gave a hint that GPC3 is somehow involved in cancer cell proliferation. One possibility is that HS proteoglycans (HSPGs) may act as supporting receptors or storage for growth factors such as Hedgehogs, bone morphogenetic proteins, and fibroblast growth factors (FGFs). A various amount of evidence supports this point of view, where sulfatase 2 (SULF2) was elevated in breast cancer and HCC. For elaboration, sulfatase 2 enzyme cleaves HSPGs at 6-O-sulphate site. Also, SULF2 knockout decreased tumorigenesis of pancreatic cancer cell lines. A study investigated the SULF2-GPC3-Wnt signaling triad. It concluded that sulfatase 2 cleaves GPC3/Wnt complex at the HS region, therefore releasing Wnt protein to initiate signaling cascade (**Figure 3**). Interestingly, GPC3 can interact with Wnt molecules independent of HS chains and accelerate cancer cell division by activation of canonical Wnt signaling pathway. These results indicate that GPC3/Wnts complex acts as a growth factor by binding to other proteins (coreceptors for either GPC3 or Wnts) (18, 86, 87). On the other hand, GPC-3 is possibly a tumor suppressor where ectopic expression increased apoptosis in A549 and NCI-H460 cell lines (88).

#### Expression Level

GPC3 gene is expressed in a time-specific manner, where it reaches peak expression during development, followed by gradual decline after birth, thus its expression is down regulated in normal tissues (86). GPC3 is uniquely over-expressed in HCC with poor prognosis correlation in late stage (89, 90). It is also over expressed in oral, colorectal and ovarian cancers (91–93). Controversially, GPC3 gene hyper methylation is observed in adult cancers, which supports argument GPC3 is under expressed in mesothelioma and neuroblastoma (94, 95).

#### *In Vivo* Models 

The first antibodies against GPC3 were of murine origin, and they did not advance for therapeutic use due to immunogenicity risk. Instead, they are currently evaluated for their use in assaying GPC3 in serum and tissue (i.e., diagnostic use) (12, 86). The first therapeutic mAb against GPC3, called GC33 (IgG2a, κ) was reported in 2008. Its epitope is located near the C-terminus (residues: 524–563). It was raised in mice and effectively induced antibody-dependent cellular cytotoxicity (ADCC) against subcutaneously transplanted HepG2 and HuH-7 xenografts (12, 86). Accordingly, GC33 was modified by recombinant technology yielding humanized GC33 (hGC-33/codrituzumab) (12, 86). Interestingly, Hep-G2 expressed higher levels of GPC3 compared to Huh-7 based on fluorescence-activated cell sorting (FACS) analysis, where Hep-G2 expressed 1.5x10^6^ GPC3 molecules per cell, while HuH-7 expressed 4.0 x 10^4^ GPC3 molecules per cell. These findings are extrapolated *in vivo*, where Huh-7 mice xenograft models demonstrated significant tumor reduction from 3000 mm^3^ to 1500 mm^3^ when treated with 1mg/kg GC33, and to 1000 mm^3^ when treated with 5 mg/kg GC33, compared to untreated control. Similarly, Hep-G2 xenograft models demonstrated significant tumor reduction from 1200 mm^3^ to 600 mm^3^ when treated with 1mg/kg GC33 and to 100 mm^3^ when treated with 5 mg/kg GC33, compared to untreated control. Testing the efficacy of sorafenib/GC33 combination on Hep-G2 xenograft model revealed that, indeed, tumor volume was significantly decreased from 1000 mm^3^ to 200 mm^3^ when treated with 1 mg/kg GC33 plus 80 mg/kg sorafenib, compared to untreated control. Meanwhile, sorafenib and GC33 had equal tumor reduction ability with tumor volume reduced to 450 mm^3^. The study also evaluated the efficacy of doxorubicin/GC33 combination on Huh-7 xenograft model. This showed that tumor volume was significantly decreased from 3000 mm3 to 1000 mm3 when treated with 5 mg/kg GC33 plus 3 mg/kg doxorubicin compared to untreated control, whereas doxorubicin and GC33 also had equal tumor reduction ability with tumor volume reduced to 1750 mm^3^ (96).

#### Clinical Trials 

Currently GC33 is under clinical trials. Encouragingly, a phase-I clinical trial showed no maximum tolerated dose (MTD) because no dose-limiting toxicities (DLTs) happened. However, reported side effects included fatigue, constipation, headache, and decreased sodium level. Another ongoing clinical trial studies the combination of hGC33 with sorafenib. Meanwhile, a phase-I clinical trial consisting of advanced HCC patients showed that murine GC33 can be safely administered intravenously up to 20 mg/kg weekly (97, 98).

In addition, a GPC3 peptide vaccine was reported to induce CD8+ activity in HLA-A2.1 transgenic mice without causing autoimmune side reactions, where treatment of NOD/SCID mice with the cytotoxic T-lymphocytes (CTLs) significantly inhibited the growth of human HCC xenografts. Therefore, phase-I clinical trials were initiated, with the vaccine consisting of two GPC3-derived peptides and an incomplete Freund’s adjuvant in advanced HCC patients. Spectacular results were achieved as the vaccine was well tolerated and 30 out of 33 patients demonstrated a significant quantified immune response. Furthermore, a correlation between the intensity of immune response and overall survival was observed. Consequently, the vaccine has advanced to phase-II clinical trials and has also been evaluated in combination with chemotherapy (99). For details about clinical trials, see **Table 2**.

**Table 2:** Glypican-3 clinical trials.

NCT Number	Conditions	Interventions	Results
NCT02748837	Solid Tumors	ERY974 Phase I	Just completed Aug 2019
NCT02395250	HCC	anti-GPC3 CAR T	Just completed Aug 2019
NCT02723942	GPC3 Positive HCC	CAR-T cell immunotherapy	No data update since 2017
NCT00976170	HCC	RO5137382 (GC33) and sorafenib (Phase I b) Codrituzumab	*Drug limiting toxicities were: grade 3 hyponatremia and hypona hyperglycemia*MTD for GC-33/sorafenib combination was 1600 mg q2w and 400 mg bid.
NCT01507168	Metastatic HCC	RO5137382 (GC33) VS. Placebo (Phase 2)	*Median PFS in the codrituzumab vs. placebo groups were: 2.6 vs. 1.5 (hazard ratios 0.97, p=0.87), in months*Median OS was 8.7 vs. 10 (hazard ratios 0.96, p=0.82).

OS, overall survival; PFS, progression-free survival; SE, side effects; MTD, maximum tolerated dose.

#### Insights 

As per methylation analysis performed by Boily et al. (95), where methylation abnormalities were present only in female neuroblastoma samples (loss of methylation) and mainly in male WT samples (gain of methylation) (95). Therefore, these results suggest that DNA methylation of the promoter region is not essential for the transcriptional repression of the GPC3 gene and that the methylation observed in females is probably linked to the inactive X chromosome. Other possible regulators are micro-RNAs, where MiR-219-5p targeted GPC3 and inhibited HCC cell line proliferation (100). GPC3 is an attractive target for immune-based therapy due to its high expression in HCC and especially that GPC3 was found in cancerous liver cells but not normal ones (89).

### Mesothelin (MSLN)

#### Structure and History 

Mesothelin is a glycosylphosphatidylinositol (GPI) fixed cell surface protein. The human MSLN is a ~71 kDa antecedent protein of 622 amino acids, which is separated by furin into 31 kDa N-terminal megakaryocyte potentiating factor (MPF) and 40 kDa mature mesothelin attached to the cell surface (101) (**Supplementary Figure 3**). MSLN was discovered in the mid-1990s, in an investigation initiated by Ira Pastan and Mark Willingham (National Cancer Institute, NIH) aiming to find new therapeutic target to treat ovarian cancer, where they started by screening for novel antibodies that target proteins significantly overexpressed in cancer cells compared to normal cells (102). Then, different mAbs were produced by Hybridoma technology (102). Candidate mAbs were evaluated by immunohistochemistry, bringing about the revelation of mAb-K1 in 1992 (102). Since mAb-K1 binding protein is expressed in typical mesothelial cells, it was called mesothelin, where it was the first characterized 125I fractionation and phospholipase-C treatment. MSLN was also identified by western blot, with molecular weight 40 kDa, present in both OVCAR3 and Hela cells. Consequently, the K1 mAb was employed to screen a lambda cDNA library containing Hela cells genome. MSLN cDNA encoded a 69 kDa protein, which, when transfected in 3T3 cells, caused a noteworthy 40 kDa band and a minor 69 kDa band to be distinguished showing that the 40 kDa band was derived from a larger parent protein (102).

#### Pathway and Function 

**Figure 4:**
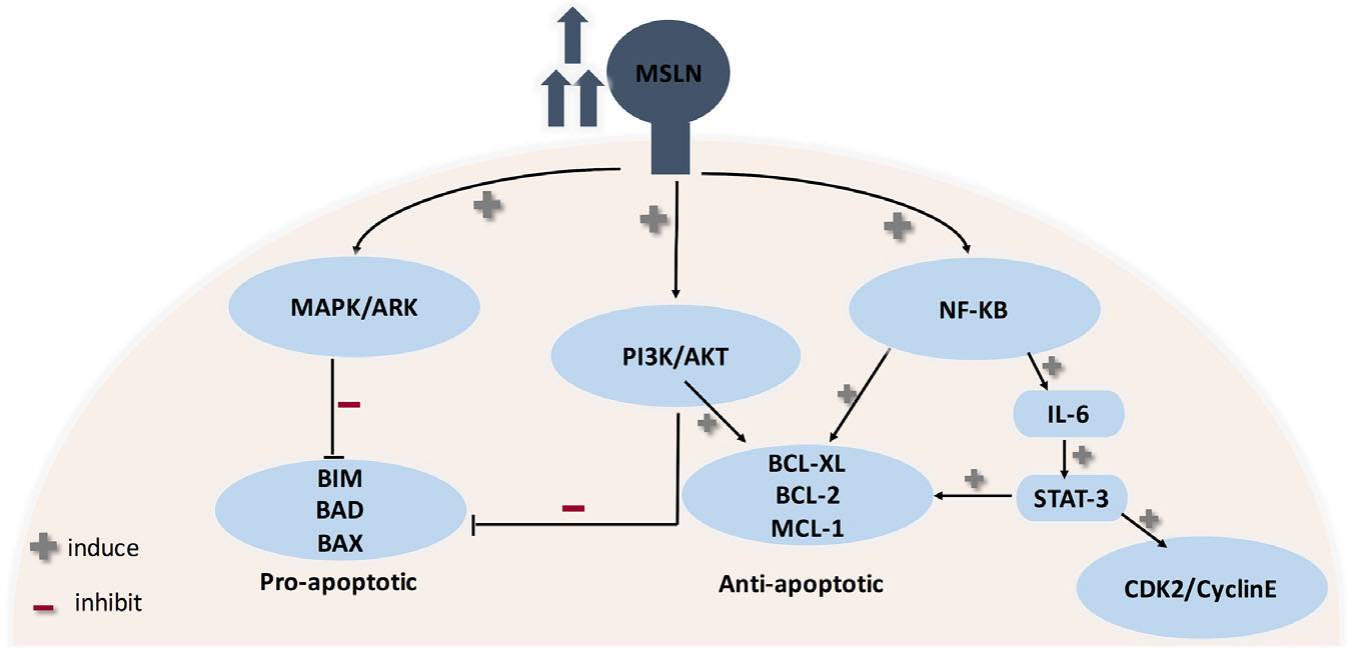
Role of MSLN in cancer progression. MSLN elevation activates MAPK/ERK, PI3K/Akt pathways therefore making cancer cells resistant to apoptosis. Also, MSLN overexpression induces NF-kB which leads to higher IL-6 production. High IL-6 induces the transcription protein 3 (Stat3), resulting in increased expression levels of the cyclin E/cyclin-dependent kinase (CDK2) complex, therefore speeding the G1-S transition resulting in enhanced cell proliferation.

The biological role of mesothelin is still anonymous because mesothelin knockout mice do not show a significant phenotypic change (12, 103). Therefore, investigations were principally done on ovarian cancer and pancreatic cancer cell lines (12). MSLN plays a pivotal role in cancer cell survival/proliferation by NF-kB activation which induces IL-6 expression. IL-6 acts as a growth factor via a new auto/paracrine IL-6/sIL-6R signaling pathway (104). In addition, MSLN enables cancer cell survival, despite inflammation, due to resisting TNF-α-induced apoptosis, through elevating Akt/PI3K/NF-kB and IL-6/MCL-1 axes (12, 101, 103). Studies on ovarian cancer cell lines showed that mesothelin is involved in tumor adhesion and metastasis based on its binding to MUC16 (also known as CA125), due to its rich glycosylation, where O-linked and N-linked MUC-16 oligosaccharides triggered heterotypic cell adhesion (103). It was recently found that immune-reactivity against mature MSLN involved IFN-γ, IL-2, and IL-7 and was positively correlated with the survival of secondary brain-cancer patients (105). An interesting finding states that MSLN is specifically increased in CCA while Glypican-3 is specifically increased in HCC, therefore shedding light on their use as diagnostic markers differentiating HCC from CCA (12) (see **Figure 4**).

#### Expression Level 

In addition to its protumorigenic role described above, mesothelin is a valuable target for various immunotherapeutic strategies due to its high presence in ovarian, uterine, and pancreatic cancers. Recent papers show that it is also elevated in triple-negative breast cancer (TNBC)—in addition to the possession of a highly immunogenic region (Region I) (12, 103, 106, 107).

#### *In Vivo* Models 

To compare the uptake of mAb-K1 in cancer and normal cells, mice were subcutaneously injected with human mesothelin-expressing tumors and then treated with Indium-labeled mAb-K1. The results were promising and, accordingly, antitumor activity was tested by conjugating mAb-K1 to Pseudomonas exotoxin A (PE). This was the first immunotoxin, named K1-LysPE38QQR, and indeed it showed an excellent *in vitro* and *in vivo* activity. However, K1 antibody has a low affinity for mesothelin; therefore, mice were immunized with a mesothelin-expressing cDNA construct, and produced antibodies were isolated by phage display. The new antibody SS was produced. The low affinity of SS was overcome by mutagenizing CDR3 residues of the heavy chain of the Fv using hotspot mutagenesis method, increasing Kd to about 1 nmol/L. This was called SS1. SS1 Fv was fused to a PE fragment producing the recombinant immunotoxin SS1P. SS1P kills cells by binding to mesothelin, followed by endocytosis, and inactivating elongation factor 2, then halting protein synthesis, and initiating programmed cell death (12, 102, 103).

Another example is MORAb-009 (amatuximab), a recombinant humanized antibody consisting of murine SS1 Fv and human constant region (IgG1κ). *In vitro* studies illustrated that MORAb-009 kills cancer cells by ADCC and blocking mesothelin/MUC-16 interaction. *In vivo* investigations combining amatuximab with chemotherapy showed significant growth deceleration of mesothelin-positive tumors in nude mice compared to chemotherapy or MORAb-009 treatment alone (102). Another investigation was performed using pancreatic cancer xenograft model, where administration of 200 mg/kg amatuximab lowered tumor volume by 75% and tumor mass by 6 folds. The study was extended to test amatuximab efficacy as combinatorial therapy with gemcitabine. Indeed, a synergistic effect was observed, where when combined with gemcitabine, tumor mass and tumor volume were reduced to 200 mg and 400 mg. Gemcitabine reduced tumor to 600 mg and 1750 mm^3^ compared to untreated control (600 mg, 1200mm^3^) (108).

Continuous developments in antibody production have led to the discovery of the first human antibody against MSLN. It is designated as m912. M912 is believed to kill cancer cells by ADCC. In another study, a high-affinity human single-chain Fv (named HN1, unique against mesothelin) was obtained from a naïve human single-chain Fv (scFv) phage display library. To assess the therapeutic potential of HN1, a fully human IgG1κ and immunotoxin (HN1 scFv + Pseudomonas exotoxin) were produced. The HN1 demonstrated a very strong ADCC and also blocked MSLN binding to MUC-16, while HN1 immunotoxin acted similar to SS1P (12, 102).

BAY 94-9343 is an antibody drug conjugate consisting of humanized immunoglobulin G1 anti-mesothelin mAb and maytansine derivative DM4 (a tubulin polymerase inhibitor) while MDX-1382 MED2460 (Medarex) consists of MSLN mAb conjugated to duocarmycin (a DNA-alkylating agent). Antibody drug conjugate (ADC) is based on the concept that the drugs will be released into the cytoplasm after antibody drug conjugate endocytosis. Interestingly, in vitro studies of BAY 94-9343 showed that it was able to kill adjacent MSLN-lacking tumor cells with no effect on quiescent cells; a possible explanation is multiple targeting as tumor cells express heterogeneous antigens. ADCs are still in phase-I clinical studies (102, 109, 110).

#### Clinical Trials

Immunotherapy targeting MSLN includes the use of tumor vaccines, antibody-based therapies, and adoptive T-cell therapy (CAR-T cells), some of which have demonstrated outstanding results in early clinical studies. However, antibodies targeting region I do not inhibit cancer cell proliferation. Furthermore, obtaining mAbs targeting MSLN domains outside region I is a difficult task. This calls for the possibility of controlling MSLN expression through genetic targeting either by miRNAs or by CRISPR/CAS (12, 103).

To evaluate SS1P efficacy and safety, phase-I clinical trials were conducted by administering SSIP intravenously; the dose-limiting side effect was pleuritis, and unfortunately 90% of patients developed neutralizing antibodies against toxin part. Neutralizing antibodies problem was tackled by immunosuppressive regimen of pentostatin and cyclophosphamide. An interesting approach is to use recombinant technology to deduce and remove B- and T-cell epitopes in SS1P, therefore decreasing its immunogenicity. A new immunotoxin resulted from deletion of PE domain II and six residues in domain III substituted with alanine. Loss of domain II rendered the new immunotoxin small and was rapidly filtered by the kidney. This was solved by replacing the Fv with a Fab to make RG7787. Interestingly, results obtained from RG7787 were promising, where large doses were safely administered to mice, with a lower risk of capillary leak syndrome in rats, and it has significant antitumor activity in mice bearing several types of mesothelin-positive tumors. Consequently, RG7787 advanced to Phase-I clinical trials (102, 109–112).

Another drawback was the large size coupled with relatively short life in circulation (20 min in mice and 2–8 h in humans), which resulted in poor penetration into solid tumors. A possible solution is combination with chemotherapy, where preclinical studies showed that chemotherapy relaxes tumor cell compact structure and lowers intratumoral fluid pressure, therefore allowing immunotoxin to reach more cells within the tumor, and produce better antitumor responses. Using confocal microscopy, SS1P penetration was studied in 3D tumor mesothelioma spheroids. Sensitivity to SS1P of spheroids was 100 times lower compared to primary cell lines grown as monolayer. This finding was explained by significant rise in the count of tight junctions inside the spheroids, with specific elevation of E-cadherin gene. This was supported by enhanced SS1P immunotoxin therapy in vitro when combined with small interfering RNA silencing and antibody inhibition targeting E-cadherin (102, 109).

Phase-I clinical trials proved the safety of amatuximab, where maximum tolerated dose was established as 200 mg/m^2^. Unfortunately, a randomized phase-II clinical trial of amatuximab/gemcitabine combination failed to show additive advantage in pancreatic cancer population compared to gemcitabine alone. However, in a nonrandomized clinical trial involving advanced unresectable pleural mesothelioma patients, amatuximab was co-administered with pemetrexed and cisplatin. Results showed improvement in overall quality of life, despite failure in increasing progression-free survival as compared with historical controls (102, 109, 113–115).

Based on the promising results obtained from MSLN mAbs, immunotoxins, and ADCs, there is a growing interest in exploring MSLN as a target for CAR-T cell therapy, where T-cells are manipulated to produce variable chain/T-cell receptor hybrid so that cancer cell is recognized by CAR-T cell binding to the tumor antigen, which activates T-cell signaling and results in cancer-cell killing. Currently, there are two approaches for anti-mesothelin CAR-T cells synthesis: either direct treatment with antimesothelin modified lymphocytes or autologous redirection (where patients’ T-cells are edited for Fv against MSLN and then returned back) (53, 59, 66).

The feasibility of MSLN CAR T-cell therapy was evaluated *in vivo* using subcutaneous or orthotopic mouse models of mesothelioma, ovarian cancer, and lung cancer, where local administration of CAR-T cells produced better results due to earlier antigen recognition, which is reflected as increased CD8+ CAR-T cell proliferation and function. Therefore, various clinical trials for MSLN CAR-T cells were started either alone or combined with chemotherapy to determine the safety and the maximum tolerated dose. A major challenge limiting the safety of CAR-T therapy is on-target/off-tumor toxicity, which is tackled by transfection of mRNA that encodes MSLN CAR into patients’ T-cells and then returning it back (i.e., autologous). Indeed, this idea demonstrated positive results in preclinical models and therefore advanced to clinical trials’ stage. The only problem for this method is that the effect is transient, lasting only for few days. Luckily, a clinical trial involving multiple infusions of MSLN CAR-T cells (SS1–4-1BB) was safe without toxicity observed. Even better, MSLN CAR T-cell therapy caused transient elevation of inflammatory cytokines in the sera, including IL-12, IL-6, G-CSF, MIP1β, MCP1, IL1RA, and RANTES. Consequently, tumor lysis and inflammation occurred, which led to the release of multiple antigens, stimulating immune response. As a result, a polyclonal IgG antibody response was detected. This phenomenon is called epitope spreading. Unfortunately, only one patient suffered from a severe anaphylactic shock and cardiac shock due to high production of IgE against MSLN CAR-T cells (102, 109, 116, 117).

An alternative strategy is increasing T-cell safety through a suicide gene to remove T-cells as soon as an adverse event occurs, such as drug-induced activation of a suicide gene, for example, the herpes simplex thymidine kinase (HSV-TK) gene, inducible caspase-9 (iCaspase-9), or EGFRΔ gene. This concept of “safety-switch systems” has succeeded in clinical investigation and is currently put under clinical trial. Preclinical studies proved the safety of suicide gene in CAR-T therapy where a single dose of the AP1903 small molecule (clinical-grade construct with an iCaspase-9 safety switch) successfully removed MSLN CAR-T cells at the peak of their proliferation. Furthermore, a particular concern regarding MSLN CAR-T cells is cross-reaction with soluble MSLN related peptide (SMRP), which could occupy and block the scFv portion, therefore causing loss of CAR-T cell activity, especially with MSLN and SMRP having identical sequence. Reassuringly, MSLN CAR-T cell activation (cytokine secretion and cytotoxic activity) depends on MSLN attached to the cell surface, where the presence of serum SMRP at high level did not alter MSLN CAR-T cell efficiency neither in vitro nor in vivo (102, 118).

Ultimately, the sole mesothelin vaccine now in clinical trials is CRS-207. It is a live attenuated vaccine produced using *Listeria monocytogenes* vector that overexpresses human MSLN. So far, the suitability of this immunization was assessed in stage-I clinical trials in patients with mesothelin-positive-resisting tumors. A stage-II clinical trial comprised of advanced pancreatic cancer population yielded noteworthy outcomes, where patients were administered either six cycles of GVAX (allogeneic pancreatic cell lines secreting granulocyte macrophage colony stimulating factor) alone or two rounds of GVAX followed by four cycles of CRS-207 every 3 weeks. After a median follow-up of 7.8 months, the median overall survival of patients treated with GVAX/CRS-207 was 6.1 months versus 3.9 months for patients treated with GVAX alone (*P*=0.011). However, this study included just 90 patients. Subsequently, approval in a larger stage-III setting is required (102, 119, 120). For a summary of MSLN immunotherapies, see **Table 3**.

**Table 3:** MSLN targeted therapy in clinical trials.

NCT Number	Conditions	Interventions	Results
NCT00570713	Pancreatic Cancer	**Phase 2:** • MORAb-009 • Placebo • Gemcitabine	**mAb-009+Gemcitabine** 1-OS=6.5 mon. (4.5 to 8.10) 2-AE=67.12% **Placebo+Gemcitabine** 1-OS=6.9 mon. (5.4to 8.8) 2-AE=72%
NCT01018784	mesothelin-positive solid tumors	MORAb-009 (Phase 1)	1-MTD=200 mg/m2 2-AEs=76.5% grade 1 fatigue and pyrexia
NCT00006981	Mesothelin positive tumours	• SS1P	1-MTD = 25 microg/kg/d ×10 2- Serious AE: 1/6 reversible vascular leak syndrome 3-all patients developed antibodies against SS1P
NCT01362790	• Mesothelioma • Adenocarcinoma of Lung • Pancreatic Neoplasms	• SS1P • Pentostatin • cyclophosphamide	**For SS1P then pento** 1- Response Assessment: 18.2% partial response, 45.5% stable disease,27.3% progressive 2-DOR = 16.3mon. (10.6 to 26.2) 3-OS= 11.8 months(1.6 to 13.6) **For pento then SS1P** 1- Response Assessment: 75% stable disease,12.5%progressive 2-OS = 8.8 mon. (0.6 to 13.0)
NCT01355965	Malignant Pleural Mesothelioma	autologous transfected anti-mesothelin CAR T cells	• 21 infusions were administered • 1 patient had serious AE • 1 patient had minimal arthralgias and fatigue
NCT01417000	• Metastatic Pancreatic Cancer	• GVAX Vaccine (CRS-207) • Cyclophosphamide	**For Cy/GVAX + CRS-207:** 1-OS = 6.28 months (4.47 to 9.40) 2-Serious AE: 29/64 **For Cy or GVAX **alone: 1-OS = 4.07 months (3.32 to 5.42) 2-Serious AE: 10/29
NCT02004262	• 2nd-line, 3rd-line and greater metastatic pancreatic cancer	• GVAX Vaccine • CRS-207 • Gemcitabine • Cyclophosphamide	**For Cy/GVAX + CRS-207** 1-OS = 3.8 months (2.9 to 5.3) 2-Serious AE: 44/94 **For CRS-207** 1-OS=5.4 months (4.2 to 6.9) 2-Serious AE: 32/87 **For Gemcitabine** 1-OS=4.6 months (4.2 to 5.8) 2-Serious AE: 15/52

OS, overall survival; PFS, progression-free survival; SE, side effects; MTD, maximum tolerated dose.

#### Insights 

MSLN immunotherapy is the most promising among GPI-AP immunotherapy, with variety of models currently tested in clinical trials. However, further studies are required for its use as biomarker to decipher its sensitivity and specificity. Currently, SMRP and MSLN are recommended in combination with other serological biomarkers. Surprisingly, significant MSLN promoter hypo-methylation was observed in mesothelioma and lung cancer patients with previous asbestos exposure. However, no correlation was found between MSLN hypo-methylation and SMRP serum levels. These findings do not only account for MSLN over expression in multiple tumors, but more importantly they support MSLN regulation by other epigenetic methods. For instance, our investigation proved that miR-2355 targeted PIG-C and MSLN mRNAs (121). Another example is MSLN targeting by miR-21-5p that decreased MERO-14 cells proliferation (122). Other examples are miR-611 and miR-877 that were tested in mesothelioma cell lines. Astonishingly, miR-611 could not degrade MSLN harboring SNP rs1057147 (123).

## Practical Implications

### Immune Painting

A rising star in targeted immunotherapy is increasing the levels of costimulatory proteins within cancer cells in order to reverse immune suppression. This can be achieved by attaching GPI anchor sequence to costimulatory proteins such as CD80, ICAM-1, and CD86, which are originally Type-I transmembrane protein. The GPI anchor is obtained from naturally occurring GPI anchoring proteins. This is called immune painting. There are two options for immune surface painting, either genetic engineering or protein engineering. Genetic engineering is construction of plasmid consisting of the DNA sequence coding for the GPI anchor attached to the sequence encoding the protein of interest, whereas protein engineering means that GPI-modified protein is directly incorporated into the cell membrane; this is based on the fact that GPI-anchored proteins can be exchanged between cells. Both strategies have shown amazing results in terms of elevating immune response; plasmids are being studied as DNA vaccine and possible use in oncolytic virus therapy, whereas protein engineering is used in cases where limited biochemical information renders specific enrichment difficult, for example, in case of enveloped viruses (124).

### Noninvasive Tests

An interesting thought is to benefit from GPI-APs in noninvasive tests, which assay binding of membrane GPIs to enzymes such as alpha-toxin and aerolysin upon cell lysis (125–127). An interesting thought for MSLN is to assay SMRP (MSLN shed in serum, identical sequence) using ELISA; this is possible because MSLN overexpression is directly reflected in serum as elevated SMRP (102, 128–131). Indeed, this rationale can be extended for GPI-shed proteins such as serum CD55, serum CD59, and serum Glypican-3. Together, these features shed light on new noninvasive tests. An outlaw, however, is the utility of Glypican-3 in the diagnosis of HCC, where it is found to indicate poor differentiation, and, is therefore not useful as an early biomarker.

### Chemical Synthesis

A challenging task in the attempt to study the structure activity relationship (SAR) of GPI-anchored proteins was the chemical synthesis of GPI anchor that started in the late 90s–early 2000s, especially after the discovery of VSG in 1988 by Ferguson et al. Simply, GPI building blocks (mannose, glucosamine, inositol, and phospholipids) are connected together through a series of chemical reactions. However, there are some obstacles. First of all, the chirality of molecules is critical for GPI-AP protein function. Moreover, oxidation and reduction complexity affect the lipid tail. Such drawbacks were tackled by the use of modern blocking methods rather than global blocking (15, 132). This resulted in the successful synthesis of naturally occurring GPI-AP and semisynthetic/synthetic derivatives containing unsaturated lipids (e.g., click chemistry tags), or highly branched structures (132). In fact, a chemical method, designated one pot ligation (OPL), was used to achieve semi-synthetic GPI anchored eGFP, Thy1, and the Plasmodium berghei protein MSP1_19_. Interestingly, GPI attachment did not cause a change in peptide structure, but it resulted in a strong inflammatory response in vitro (133).

## Overall Insight

**Figure 5:**
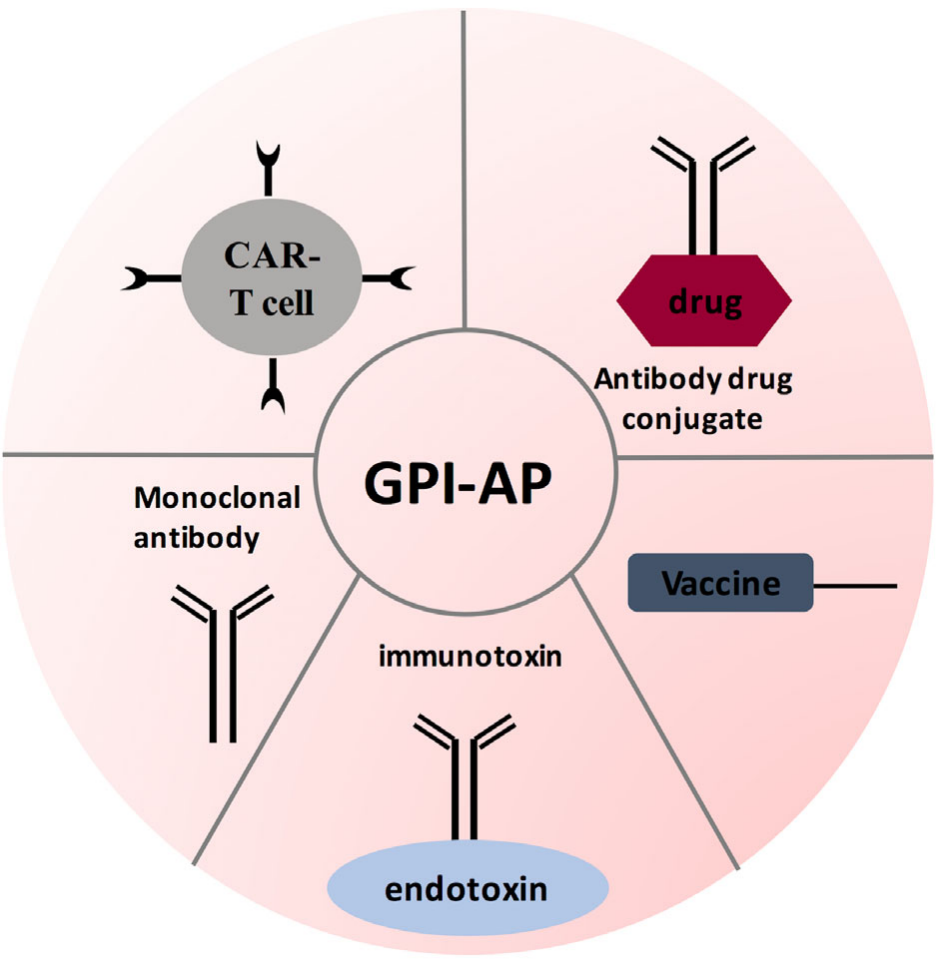
GPI-AP-targeted immunotherapy strategies. Several therapeutic strategies have been designed for targeting GPI-AP on tumor cells: tumor vaccine strategy antibody-based therapies; and adoptive CAR T- cell therapy. These therapies are being evaluated in phase I and/or phase II clinical trials.

To conclude, GPI-APs are necessary for various biological functions. Advancement in oncology research has shown that GPI-anchored proteins play a critical role in cancer progression. Further studies demonstrated that GPI-anchored proteins have a unique expression pattern on neoplastic cells. Consequently, scientists are trying to use GPI-APs for early diagnosis and targeted therapy. Throughout this review, we focused on few GPI-anchored proteins (MSLN, Glypican-3, CEA, DAF, and MAC-i), with these proteins overexpressed in many cancers. When used as biomarkers, GPI-APs demonstrated high specificity. However, new assaying technologies are needed to enhance GPI-APs sensitivity. Another opportunity is investigating the expression level of GPI pathway members in different cancers. Surprisingly, our work showed that PIG-C expression is elevated in TNBC, while PIG-U expression was down regulated in KRAS mutant CRC (29, 30). This raises a critical question: why GPC-3 and CEA are over expressed in CRC? This opens the door for investigating whether other GPI pathway genes are elevated in CRC to compensate for PIG-U loss. Next, the status of immune-based therapies (antibodies, ADCs, CAR-T therapy, and tumor vaccine) was reviewed (see **Figure 5**). Encouragingly, many therapies advanced to clinical trials. Indeed, GPI-AP immunotherapies have improved response rate, overall survival and progression free survival when combined conventional chemotherapies. However, when testing GPI-AP immunotherapies alone, clinical trials results were controversial, where some studies showed significant improvement in response rate compared to control group (treated with standard chemotherapies), while other studies failed to show significant response rate improvement. These findings surely call for continuous research in this field, with the possibility of trying other therapies like genetic targeting with siRNAs and CRISPR/CAS, especially with expansion of non-coding RNAs targeting GPI-APs. As we mentioned; miR-219 targeted GPC-3, while miR-21, miR-611 and miR-877 targeted MSLN (100, 122, 123). In addition, our recent work showed that miR-2355 targeted PIG-C and MSLN mRNAs (121). PIG-C manipulation has also significantly impacted MSLN surface level (121). Another example is the success of CRISPR-Cas9-engineered mouse model for GPI anchor deficiency to resemble human phenotype (134). Additionally, PIG-V knock-out resulted in hippocampal synaptic dysfunctions (134). These findings inevitably prompt the utilization of RNA interference therapy to target GPI pathway genes as well as GPI-APs in cancer, whereby blocking GPI pathway can be a promising therapy as multiple signaling pathways will be cut off, causing death of cancer cells.

## Author Contributions

All authors contributed to the article and approved the submitted version.

## Conflict of Interest

The authors declare that the research was conducted in the absence of any commercial or financial relationships that could be construed as a potential conflict of interest.
