# Supplementary material for: GPI-AP: Unraveling a New Class of Malignancy Mediators and Potential Immunotherapy Targets
Source: Front Oncol. 2020 Dec 4;10:537311. doi: 10.3389/fonc.2020.537311 (PMC7746843; doi:10.3389/fonc.2020.537311)
Supplement: Supplementary file 1 [file DataSheet_1.docx]

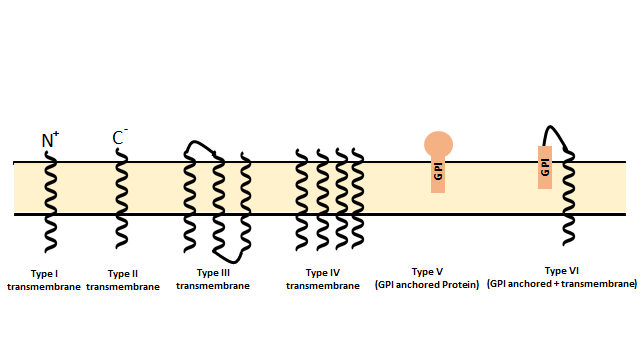


**Supplementary Figure 1: Types of transmembrane proteins**

Type I transmembrane proteins have one transmembrane helix with N-termius outside. On the other hand, Type II transmembrane proteins have one transmembrane helix with C-termius outside. Type III transmembrane proteins are characterized by a single polypeptide chain spanning the cell membrane multiple times. Meanwhile, Type IV transmembrane proteins are characterized by having separate polypeptide chains. In addition, GPI anchored proteins (Type V) are proteins covalently bound to lipid. Finally, Type VI is a combination of transmembrane helix and covalently bound lipid. Another group of membrane proteins are peripheral proteins which consist of a polypeptide chain that remotely interacts with other transmembrane proteins (eg.H-bonds)


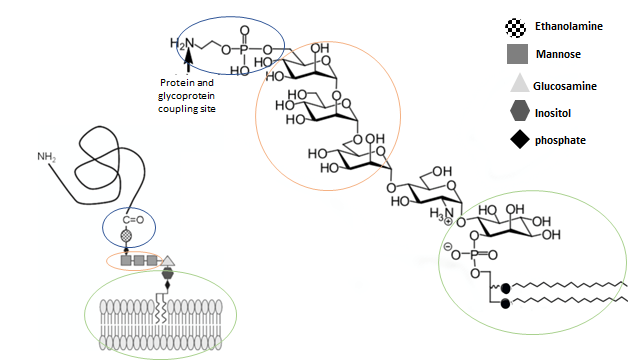


**Supplementary Figure 2: structure of GPI anchored protein** The glycosyl-phosphatidyl-inositol (GPI) anchor is a posttranslational modification that attaches the modified protein to the outer leaflet of the cell membrane. The GPI anchor is a complex structure consisting of phospho-ethanol-amine linker, glycan core (Inositol+ Glucosamine+ Mannose), and phospholipid tail.


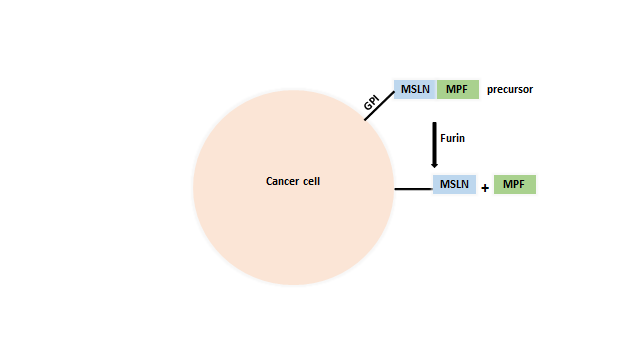


**Suuplementary Figure 3: structure of Mesothelin.** Precursor MSLN is a 71 KDa protein consisting of 622 amino acids. The N-terminal signal peptide (residues 1–33) and the C-terminal anchor addition signal (at predicted cleavage site: Ser598) are removed. C-terminal anchor addition signal is then replaced with a GPI anchor. The MSLN precursor is cleaved by furin at Arg295 into two products, a ~31kDa mature megakaryocyte potentiating factor (MPF) and a ~40kDa GPI-anchored membrane-bound mature mesothelin. Interestingly, the structure of MSLN is still unknown where in silico analysis predicts that the structure model for mesothelin is made of tandem repeats of approximately 50 residue-long helix-turn-helix motifs and that the cleavage does not change the 3D confirmation of Mesothelin

**Supplementary Table 1. Proteins involved in biosynthesis of GPI-APs. (Reference 7,8)**

| Step | Enzyme | Genes/Protein subunit |
| --- | --- | --- |
| 1 | GPI-GlcNAc transferase (GPI-GnT) | PIG-A PIG-C PIG-H  PIG P PIG-Q PIG-Y DPM2 |
| 2 | GlcNAc-PI de-N-acetylase | PIG-L |
| 3 | Flippase | Not applicable |
| 4 | Inositol acyltransferase | PIG-W |
| 5 | a1-4mannosyltransferase I (GPI-MTI) | PIG-M (catalytic)  PIG-X (stabilization) |
| 6 | a1-6mannosyltransferase II (GPI-MTII) | PIG-V |
| 7 | EtNP transferase I (GPI-ETI) | PIG-N |
| 8 | a1-2mannosyltransferase III(GPI-MTIII) | PIG-B |
| 9 | EtNP transferase III (GPI-ETIII) | PIG-O (catalytic)  PIG-F (stabilization) |
| 10 | EtNP transferase II (GPI-ETII) | PIG-G (GPI7)  PIG-F |
| M4 (mannose addition) | a1-2mannosyltransferase IV (GPI-MTIV) | PIG-Z (SMP3) |
| TA (Trans-amination) | GPI transamidase | PIG-K GPAA1 PIG-S |

|  |  | PIG-T PIG-U |
| --- | --- | --- |
| Post attachment (1) | Inositol deacylase | PGAP1 |
| Post attachment (2) |  | PGAP5/MPPE1 |
| Post attachment (3) | GPI phospholipase A2 | PGAP 3 |
| Post attachment (4) | Lyso-GPI-AP acyltransferase | PGAP 2 (non-catalytic) |

**Supplementary Table 2. different biological roles of GPI anchored proteins (References 6,7)**

| Biological role | Protein |
| --- | --- |
| Enzymes | human erythrocyte AChE  pig kidney membrane dipeptidase human kidney membrane dipeptidase bovine liver 5ʹ-nucleotidase  human placental APase |
| cell-cell interaction | mouse skeletal muscle NCAM  human LFA-3 |
| Complement regulation | Human CD 55  Human CD 59 |
| mammalian antigens | rat brain Thy-1 human CD-52 humanCD-14  human CEA |
